# Body, image, and digital technology in adolescence and contemporary youth culture

**DOI:** 10.3389/fpsyg.2024.1445098

**Published:** 2024-10-23

**Authors:** Francesco Demaria, Maria Pontillo, Cristina Di Vincenzo, Domenica Bellantoni, Italo Pretelli, Stefano Vicari

**Affiliations:** ^1^Child and Adolescence Neuropsychiatry Unit, IRCCS, Bambino Gesù Children’s Hospital, Rome, Italy; ^2^Anorexia Nervosa and Eating Disorder Unit, Child Neuropsychiatry, Department of Neuroscience, Istituto di Ricovero e Cura a Carattere Scientifico (IRCCS), Bambino Gesù Children's Hospital, Rome, Italy; ^3^Department of Life Sciences and Public Health, Università Cattolica del Sacro Cuore, Rome, Italy

**Keywords:** body image, digital technology, adolescence, youth culture, self-perception

## Abstract

The physical, psychological and social changes that occur during adolescence constitute a physiological crisis that is necessary for development and growth. The establishment of a suitable “self-image” is important for facilitating harmonious psychophysical development during this time. In the current era, digital technology (DT) serves as an extraordinary means of communication for young people, who make significant use of images as a mode of expression. Accordingly, there is growing interest in the relationship between physical development, self-image and use of DT. A review of the published literature on the topic was carried out in April 2024. Fourteen studies (*n* = 14) were inclused from search of electronic databases such as PubMed, CINAHL, PsycInfo, MedLine, and Cochrane Library. The aim of this study is to explore the influence of (DT) on cultural models of adolescent body image, and how this “social” culture can affect wellbeing and development. It was considered that the rise of DT and social media (SM) emphasized in young people the culture of appearance, adherence to ideal models (thinness ideal) and social comparison at an unprecedented level. It was estimated that the digital mechanism works on the adolescent’s vulnerability and stimulates the desire for experimentation and amplifies cultural beliefs that expose the young to deviant or pathological behaviors on the body. The use of digital images emphasizes the perception of self by making it more real and alive but empty of content. Our framework highlights that the adolescent can defend himself if he leaves the homologation that the SM condition, regains his own experiences, fill with emotional content and real life the images and the representation of the body.

## 1 Introduction

Adolescence represents a transitional phase characterized by significant physical, psychological and social changes (Rosenfeld and Nicodemus, 2003; Kinghorn et al., 2018). These changes determine a physiological crisis that promotes a new perception of one’s self and others, as well as new relationships and modes of adaptation (Martin et al., 2014; McDonagh et al., 2018; Sawyer et al., 2018). Initially, adolescents’ physical and sexual development at puberty may trigger experiences of impotence. However, as their bodies become stronger and more mature, they may develop psychosocial and interpersonal skills that enhance self-control (Kotiuga et al., 2023). Fundamental to these physiological and psychophysical changes are body modifications and the development of a new “self-image” (Bordeleau et al., 2023; Lacroix et al., 2023; Vankerckhoven et al., 2023). Body image refers to the thoughts, emotions, perceptions, and actions concerning one’s physical self and the experience of being in one’s body. It is a multi-faceted concept that includes how we perceive and feel about our bodies, as well as how we interact with and inhabit them (Keeton et al., 1990; Tylka and Wood-Barcalow, 2015; Piran, 2017).

The advent of DT as a means of fostering communication and social relationships (Vogels et al., 2022) has created an environment in which adolescents regularly upload images to social networks, in search of comments and/or social approval (Revranche et al., 2022). Their frequent acts of “capturing” their bodies and personal moments in imagery emphasizes the intensity of adolescence as a developmental phase in which recognition and self-assertion are paramount (Le Breton, 2016). Several theories proposed before the advent of the digital age, have address how socio-cultural pressures may affect the relationship and confrontation, and emphasize the physical aspect. The theory about “the tripartite influence model of body image” (Thompson et al., 1999) proposes that three socio-cultural forces (peer, parent and media) influence the body image through the mechanisms of internalization of the thin ideal (such as that of thinness), and fostering appearance comparisons, particularly of girls.

The internalization of the thinness ideal refers to the extent to which an individual internalizes culturally defined standards of beauty. Aspect comparisons are linked to Festinger’s (1954) “theory of social comparison,” which proposes that individuals engage in social comparison to estimate their own social status relative to others. Social confrontation has been linked to body dissatisfaction in boys and girls. Another fundamental theory is the “theory of objectification” (Fredrickson and Roberts, 1997), which proposes that in a culture that objectifies women’s bodies sexually, girls learn to adopt the perspective of observing and monitoring their body (self-objectification), to treat oneself as an object to be looked at and evaluated on the basis of physical appearance.

Adolescence is characterized by complex biopsychosocial changes that promote the exploration of identity, the beginning of romantic and sexual relationships and the search for new sensations.

In the context of these complex biological, cognitive and interpersonal changes, a culture of appearance develops during adolescence determines normative behaviors that reinforces the ideals of beauty; adolescents frequently discuss the physical attractiveness and how to improve it, encouraging social comparison and exacerbating physical dissatisfaction (Jones et al., 2004). Theories on SM have increasingly considered the impact on individuals’ experiences and behaviors (Moreno and Uhls, 2019; Granic et al., 2020).

Media effects scholars have argued that SM sites are made up of unique features (e.g., “24 h a day,” “7 days a week,” publicness) which impact social experiences and developmental processes of adolescents (Borzekowski and Bayer, 2005; Subrahmanyam and Šmahel, 2011; Valkenburg and Peter, 2011; Gjylbegaj, 2018). Nesi et al. (2018a, 2018b) based on previous literature, described seven SM characteristics: visualness, quantifiability, availability, publicness, permanence, asynchronicity, and cue absence. Permanence, for example, is a feature of SM that describes the permanent accessibility of content, which can stimulate the attention of adolescents on their appearance in images.

Visualness, publicness and availability stimulate people to focus on their own physical appearance and appearance of their peers; quantifiability stimulates peer feedback. Considering this, the emerging youth culture of digital communication encourages to rethink adolescence as a developmental phase constituted by cultural beliefs and ideal models of body image. The interaction between physical change and self-image during adolescence warrants in-depth exploration, particularly as it pertains to adolescents’ use of SM (Dienlin and Johannes, 2020; Revranche et al., 2022; Vankerckhoven et al., 2023). In their quest to establish a new identity, adolescents find themselves confronted and significantly influenced by social trends and cultures (Kinghorn et al., 2018).

Modern society promotes strong models of affirmation, behavioral stereotypes and ideals (Aniulis et al., 2021; Ohashi et al., 2023), which are disseminated and amplified via digital communication. Research is sensitive to cultural trends and patterns. It is possible to expect that special scientific attention will be given to the importance that body image plays today, in a development phase sensitive to the ideal of beauty and the role played by DT on the culture of appearance.

The aim of this study is to explore the influence of DT on cultural models of adolescent body image, and how this “social” culture can affect wellbeing and development.

## 2 Methodology

This study includes a review of the literature published on the topic according to PRISMA guidelines (Preferred Reporting Items for Systematic Reviews and Meta-Analyses) (Page et al., 2021). A thorough review of scholarly works was conducted, carefully verifying sources using electronic databases such as PubMed, CINAHL, PsycInfo, MedLine, and Cochrane Library. Keywords used in the search included: (“Body image” OR “Digital technology” OR “Adolescence”) AND (“Youth culture” OR “self-perception”). The review was conducted on April 30th at 10:30 a.m. and considered the period from June 2017 to April 2024.

Inclusion criteria:

- Original research articles.
- Observational articles (cross-sectional study, retrospective study).
- Experimental studies.
- Theoretical studies.

Exclusion criteria:

- Studies unrelated to the aim of the study.
- Overlapping studies between the various databases.
- No quantitative and standardized data (series of case studies).
- Articles for tool validation.

### 2.1 Study selection process

**Figure 1:**
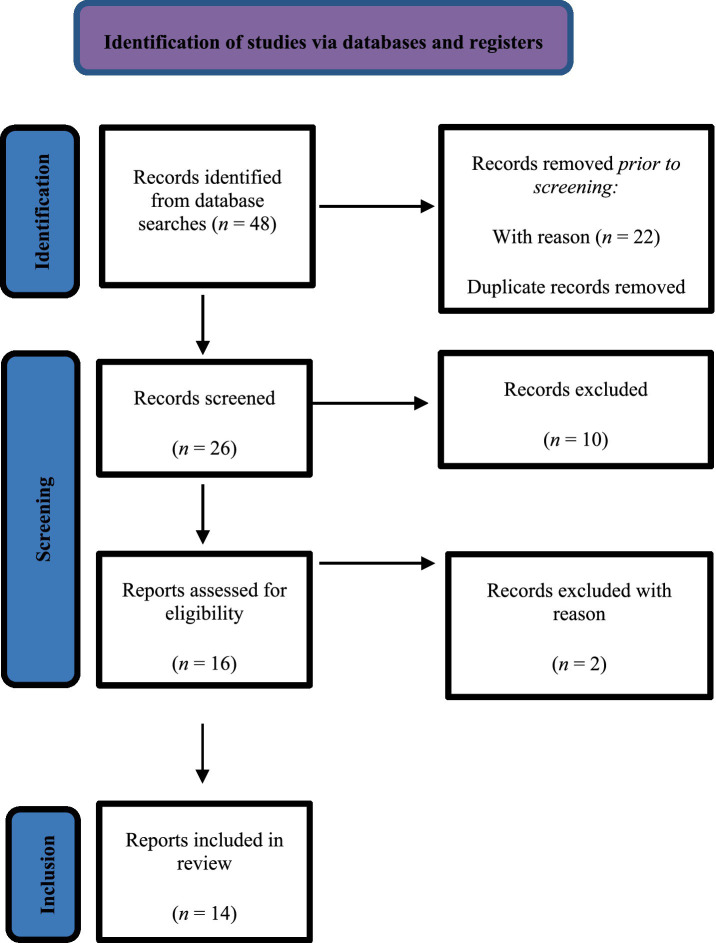
Flow chart of literature review.

After establishing the search strategy for each database, study titles and abstracts were reviewed and studies deemed unrelated were excluded. The full texts of the remaining articles were then scrutinized in accordance with the predetermined inclusion and exclusion criteria, leading to the exclusion of irrelevant studies. Finally, the articles that met all the inclusion criteria were submitted to a qualitative evaluation. No language or design restrictions were applied. Figure 1 presents a detailed flow diagram of the study selection process.

## 3 Results

The studies reviewed varied widely in their design, characteristics, and content. To address this, a narrative synthesis was performed to describe, explore, and interpret the findings, while also assessing the methodological soundness of the studies (Table 1).

**Table 1:** Describes the results of the 14 included studies.

Author(s)	Title	Study design	Summary
Aubrey et al. (2023)	Digital technologies and adolescents’ body image	Communication	Highlights both negative impacts, such as the internalization of ideals of appearance, and potential positive aspects, such as the promotion of a positive body image.
Burnette et al. (2017)	I do not need people to tell me I’m pretty on social media	Article	Explores the impact of social media on the body image of girls aged 12–14 with a total of 38 participants. Many showed strong media literacy and confidence, which helped to mitigate the negative effects on body image.
Choukas-Bradley et al. (2022)	The perfect storm: a developmental–sociocultural framework for the role of social media in adolescent girls’ body image concerns and mental health	Theoretical Review	Proposes that the characteristics of social media, such as idealized images and quantifiable feedback, intersect with developmental factors and socio-cultural processes of gender socialization to exacerbate concerns about body image.
Goodyear (2020)	Narrative matters: young people, social media and body image	Communication	Adult-oriented work to help better understand how social media shapes and affects the health and wellbeing of young people.
Holly et al. (2023)	Optimizing adolescent wellbeing in a digital age	Theoretical Review	Highlights the need to empower adolescents with digital literacy and skills, while strengthening governance mechanisms to manage risks associated with digital environments.
Krogh (2022)	The beautiful and the fit reap the spoils: body image as a condition for the positive effects of electronic media communication on wellbeing among early adolescents	Experimental study	The effects of electronic media communication (EMC) and social media on youth health and wellbeing using data from 1,843 adolescents aged 12 to 17. The study found that early adolescents tend to feel more wellbeing with higher EMC intensity, while the positive EMC relationship disappears in the condition of a negative perceptual body image.
Jarman et al. (2021)	Social media, body satisfaction and wellbeing among adolescents: a mediation model of appearance-ideal internalization and comparison	Experimental study	The impact of social media on body satisfaction and adolescent wellbeing in 1,899 Australian adolescents between 11 and 17 years old. It examines how the internalization of the ideals of appearance and the comparison with these ideals can mediate the relationship between the use of social media and body satisfaction. The results suggest that social media can negatively affect body satisfaction and general wellbeing in adolescents.
Magis-Weinberg et al. (2021)	Context, development, and digital media: implications for very young adolescents in LMICs	Review	Discusses the impact of digital media on young adolescents in low- and middle-income countries. The authors stress that cultural and contextual factors significantly influence how digital technology is accessed and used.
Mahon and Seekis (2022)	Systematic review of digital interventions for adolescent and young adult women’s body image	Systematic review	Aimed to critically assess the use of digital interventions to address a range of mental health issues, including body image in adolescents and young adults aged 10–25. 8 out of 15 interventions were effective in improving at least one body image result from the pre-post intervention; however, the effect sizes were mostly small-medium and few effects were maintained at follow-up.
Marengo et al. (2018)	Highly-visual social media and internalizing symptoms in adolescence: the mediating role of body image concerns	Experimental study	Investigates the association between time spent on highly-visual social media (HVSM), such as Instagram and Snapchat, concerns about body image, and internalizing symptoms in a sample of 523 adolescents attending classes 6–11 in northern Italy. Students who reported frequent use of HVSM (>2 h/day) reported significantly higher concerns for body image and symptom internalization than their peers who did not report any use of HVSM.
Mesce et al. (2022)	Body image concerns: the impact of digital technologies and psychopathological risks in a normative sample of adolescents	Experimental study	Analyzes the correlations between Body Image Concerns (BIC), Internet Addiction (IA) and Social Media Addiction (SMA), and between internalizing and externalizing problems in a sample of 204 participants. Significant associations between BIC and technology addictions (SMA and IA) appeared both in the total sample and in the subgroups related to gender and age.
Papageorgiou et al. (2022)	Why do not I look like her? How adolescent girls view social media and its connection to body image	Experimental study	Explores how sexualized images typically found on social media could affect the mental health of adolescent girls, and contribute to increased body dissatisfaction. In-depth interviews were conducted with girls aged 14–17 (*n* = 24) in Perth, Western Australia. Comparison of appearance was perceived as a factor aggravating the adolescent’s concerns about body image.
Perloff (2014)	Social media effects on young women’s body image concerns: theoretical perspectives and an agenda for research	Theoretical study	Proposes a model that highlights the impact of individual vulnerability, social media use, and psychological processes on body image problems.
Sagrera et al. (2022)	Social media use and body image issues among adolescents in a vulnerable Louisiana community	Experimental study	Explores the impact of social media (SM) use on body image issues (BII) among adolescents (5.070) in a vulnerable community in Louisiana. The research highlights that increased time spent on social media correlates with higher reports of BII, especially among females; while both sexes reported BII with increasing time spent on SM. A diversity of platforms was associated with an increase in BII among SM users compared to non-users: Pinterest, Reddit, Snapchat, TikTok, Twitter and YouTube.

The works examined are specifically reported. Jarman et al. (2021) in a study of 1,899 Australian adolescents between the ages of 11 and 17 years suggest that SM may negatively influence body satisfaction and general wellbeing in adolescents. The work examines in particular how the internalization of appearance ideals and comparison with these ideals can mediate the relationship between the use of SM and body satisfaction. Similarly, in a work of small sample (*n* = 24) of girls aged 14–17 years, the results showed that appearance comparison is perceived as a factor that aggravated the adolescent’s concerns regarding body image as well as influencing the adolescent to change their appearance and seek confirmation on SM (Papageorgiou et al., 2022).

Girls are certainly more conditioned by aesthetic ideals and the culture of appearance. In a theoretical review, Choukas-Bradley et al. (2022) support the role played by SM on gender culture, such as the excessive social emphasis on the physical appearance of girls, especially in a phase of adolescent development in which comparison and relationships between peers are crucial. Body image concerns may be a key mechanism underlying associations between adolescents’ SM use and mental health (e.g., depressive symptoms and eating disorders).

Other authors have instead considered the time dedicated to the use of DT as a determining factor in stimulating body image concerns. Marengo et al. (2018) reported significantly higher concerns for body image and symptom internalization in students that use of highly visual media (HVSM) >2 h/day; while Mesce et al. (2022) identify significant associations between Body Image Concerns (BIC) and technology addictions such as Internet (IA) and Social Media Addiction (SMA) in a sample of 204 adolescents. Likewise Electronic media communication (EMC) and SM influence body image and affect adolescent wellbeing in a sample of 1,843 adolescents aged 12–17 years (Krogh, 2022).

The strong online presence of young women is important for understanding how SM can influence body image perception, considering theories of communication and social psychology. Perloff (2014) examines how the interactive format and content characteristics of SM, characterized by a strong peer presence and use of visual images, can influence body image concerns through negative social comparisons and normative processes. The impact of SM on body image issues, with deleterious effects on adolescent mental wellbeing, may transcend culture and socioeconomic status, as noted by Sagrera et al. (2022), who considered a sample of 5,070 teenagers from a public school system where more than 50% of students live in poor families in Louisiana.

Conversely, the work of Magis-Weinberg et al. (2021) considers that adolescents in low- and middle-income countries are often early adopters of mobile technology and SM platforms, underscoring how cultural and contextual factors influence access and appropriation of DT. Governance mechanisms become important to manage the risks associated with digital environments, facilitate the use of digital platforms that could enable adolescents to maintain and create new social connections, use support networks to cultivate their interests, and support adolescents in building their sense of identity (Holly et al., 2023). It is also necessary to help adults understand the ways SM shapes and influences the health and wellbeing of young people (Goodyear, 2020). Indeed, there is a need for an ecological approach to the prevention of body dissatisfaction, highlighting the importance of media literacy, which can help mitigate negative effects on body image in SM (Burnette et al., 2017). Similarly, Aubrey et al. (2023) highlight evolutionary considerations of the adolescent public and the affordances of DT, examining how DT shapes and supports positive body image. Meanwhile, Mahon and Seekis (2022), in a systematic review, evaluated the current evidence on the use of digital interventions to address body image problems in adolescent and young adult women: eight out of 15 interventions were effective in improving at least one aspect of body image from pre- to post-intervention; however, the effect size was mostly small or medium, and few effects were maintained at follow-up.

The current scientific literature describes the impact of SM on internalization of ideals of appearance, body image, adult-oriented, governance mechanisms, wellbeing, body satisfaction, influence by cultural and contextual factors, digital interventions, SM addiction, internalizing symptoms, sexualized images, theoretical studies and vulnerability in selected groups and gender. The evaluated works did not adequately address the current digital experiences of young people and did not focus on adolescent development as expected. It is suggestive to develop the topic in question.

## 4 Discussion

### 4.1 Adolescent transition: a developmental perspective

The developmental period of adolescence is characterized by transition and transformation, which impose considerable psychological strain (McDonagh et al., 2018). The transition that occurs encompasses not only physical and biological change, but also important shifts in social roles (Martin et al., 2014). In the last century, the timing of these transitions has shifted, with puberty (the psychophysical process marking the onset of adolescence) now happening at an earlier age across nearly all populations, while societal milestones such as completing education, getting married, and having children are being postponed. As a result, the transition from childhood to adulthood now spans a more prolonged period (Sawyer et al., 2012; Sawyer et al., 2018). During adolescence, the process of identity formation must align with the changes that occur in the body (Vankerckhoven et al., 2023). Physical changes are most pronounced during early adolescence, while psychological and mental development take center stage during later adolescence. At the neurobiological level, the brain experiences structural transformations in both white and gray matter. The limbic region, responsible for emotional reactions, behavioral responses, and learning, matures earlier than the prefrontal cortex, which governs executive functions, impulse control, and planning. This discrepancy leads to decreased inhibition alongside heightened perception and a propensity for seeking new experiences. Furthermore, sex hormones surge and become more active, particularly affecting the limbic system, thus intensifying the drive to explore novel and stimulating experiences (Holmes et al., 2016; Romeo, 2017; Bos et al., 2018; Riedel et al., 2019). Simultaneously, physical and psychological changes during adolescence may evoke feelings of uncertainty and instability. Emotions and behaviors are not only shaped by social and cultural realities (Kinghorn et al., 2018), but also media communication, particularly through DT. Social networks and leverage technology and digital communities to cultivate a shared identity. Engagement in a social network (e.g., commenting, visiting, retweeting) may heighten adolescents comparison with peers, provide them with feedback and increase their sense of belonging to a group or digital tribe. This can help adolescents feel safe and allow them to recognize themselves more easily (Dienlin and Johannes, 2020). In their search for personal identity, adolescents tend to reproduce the characters with whom they identify, drawing on their surrounding environment and culture of consumption. In the realm of body and image, “beauty” is a modern object of mass culture, fueled by SM that accentuate the “cult of youth” (Shen et al., 2022). During adolescence, the formation of identity also relies on the individual’s capacity to assimilate their “new body” into a self-image that will be aligns with societal ideals and curturally defined (Aniulis et al., 2021; Ohashi et al., 2023). Choukas-Bradley et al. (2022) proposes that the characteristics of SM (e.g., idealized images) intersect with developmental factors (e.g., the ideals of beauty) and the culture of appearance in adolescent girls.

Beauty is conditioned from unrealistic social expectations. SM play a delicate role in shaping communication and sharing of content (i.e., images and videos) among adolescents. Body image dissatisfaction (Hosseini and Padhy, 2023) may arise when adolescents, spent time, compare themselves to images of peers online, or when they receive negative comments or disapproval regarding their weight, body shape or eating habits (Marengo et al., 2018; Hummel and Smith, 2015). Comparison of physical appearance is a factor that can aggravate the concerns of adolescents about their body image and may lead to behaviors aimed at changing the appearance and seeking confirmation on SM (Papageorgiou et al., 2022).

Negative body image can trigger a number of behaviors aimed at “improving” one’s physical appearance, such as calorie restriction, physical activity and use of different aesthetic medical procedures to “remodel” the body, all of which may be practiced to an excessive, maladaptive extent (Sarwer and Polonsky, 2016). The cultural models that SM emphasizes became uncertain and risky. The “thinness ideal” on SM has been proven to heighten adolescents’ risk of developing disordered eating behaviors (Dane and Bhatia, 2023; Mushtaq et al., 2023). Easy access to “pro-Anorexia” websites, which depict anorexia nervosa as a virtuous lifestyle (Norris et al., 2006; Simons et al., 2024), or the pursuit of ideal body shapes through surgical interventions may further exacerbate these issues (Sonmez and Esiyok, 2023). Research has than shown that negative comments targeting overweight individuals online may heighten their risk of developing pathological symptoms of depression (Soares Filho et al., 2021; Czepielewski, 2024). Dissatisfaction with one’s body and expressions of general discomfort and suffering may also increase vulnerability (De Carvalho et al., 2020) to forms of non-suicidal self-harm, such as cutting, scratching and burning (Klonsky and Muehlenkamp, 2007; Gillies et al., 2018; De Luca et al., 2023), often associated with depressive symptoms (Niu et al., 2024). Thus, body image is a significant factor contributing to adolescent wellbeing (Amaral and Ferreira, 2017; Jiménez Flores et al., 2017; Bordeleau et al., 2023) and physical health (Alipour et al., 2015). It was seen in experimental study of Jarman et al. (2021) how internalization and comparison of the ideals of appearance can mediate the relationship between the use of SM and body satisfaction. These data suggest that SM can negatively affect body satisfaction and general wellbeing in adolescents.

The characteristics of the positive body image refer to harmonious and functioning body perception, focused on self-care (Tylka and Wood-Barcalow, 2015), neutral to identity/self-worth where beauty is not the main aim; while the characteristics of the negative body image are related to a evaluations of a body centered on appearance (Piran, 2017), with attitude of control and/or modify physical aspect. These observations highlight the delicate role played by SM on body image, which stimulate in young people the culture of appearance, adherence to beauty standards and, for girls, the emphasis of body observation (self-objectification) as an object to be looked at and considered according to the physical aspect.

### 4.2 Capturing body image

A common sight is adolescents capturing themselves in quirky poses with their phones, paying particular attention to details. Typically, the resulting images are uploaded to blogs and social networks in the hopes that they will attract comments, or they are deleted if they fail to align with a desired look or meet with social disapproval. Consequently, images are innumerable and fluid—instruments of communication and evidence of one’s presence, through body and relationship with the world (Le Breton, 2016).

Lachance (2011) explored the unique relationship that “hypermodern adolescents” have with time through their extensive utilization of new technologies, which has cultivated a novel culture of communication. In traditional and modern societies, rituals and myths organize time, nurturing a “meaningful connection with temporality” for children. However, DT and cultural artifacts are reshaping and provoking a fresh relationship with time for teenagers. In a society marked by ambiguous boundaries, where genuine independence is delayed, young individuals might grasp at temporality as a means to assert their emancipation. Walter Benjamin (2022) distinguished between “life” and “experience,” defining experience as a lived encounter subject to subjective interpretation. In the fast-paced modern youth culture, adolescents must develop strategies for translating their experiences into meaningful experiences.

### 4.3 Digital world and the future

In contemporary society, the image has achieved maximal expression, through technology (Revranche et al., 2022). Adolescents are dedicating more time to online activities, such as exchanging photos and communicating with peers. Despite concerns about the potential effects of constant connectivity on adolescents’ mental wellbeing, research has produced limited and sometimes conflicting evidence (Jensen et al., 2019; Odgers and Jensen, 2020; Blanchard et al., 2023; Weigle and Shafi, 2024). Specific concerns have been voiced about adolescents’ inappropriate and distorted use of SM and the internet, with technology addictions, regard to privacy violations, cyberbullying and excessive use leading to chronic sleep deprivation, all of which may negatively impact cognitive abilities, academic performance and socio-emotional functioning (Caceres and Holley, 2023; Khalaf et al., 2023; Mesce et al., 2022). Intervention and prevention programs, as well as educational and parental support, may foster open communication with adolescents to help them responsibly navigate technology and establish appropriate limits for its use (Awad and Connors, 2023; Douglas et al., 2023; Goodyear, 2020). Empowering adolescents with digital literacy and skills means managing the risks associated with DT and establishing appropriate limits for its use (Holly et al., 2023). This prompts reflection on how this new culture of connection is replacing lived relationships, with potentially negative consequences (Lau-Zhu et al., 2023). It is not surprising that during the COVID-19 pandemic, young individuals were significantly impacted by lockdowns and other restrictions, relying heavily on virtual channels. Vall-Roqué et al. (2021) observed that the heightened use of SM platforms (e.g., Instagram, YouTube, TikTok, Twitter, Facebook) during the pandemic correlated with body image issues, diminished self-esteem, a proclivity toward weight loss, and an elevated risk of eating disorders among adolescents overall, especially among young women.

The emerging frontier of DT is generative artificial intelligence (AI), which is capable of creating various forms of content (i.e., text, audio, images, videos) that mimic human appearance and emotions (Schuengel and Van Heerden, 2023). The potential consequences of this technology on adolescents’ self-image, and body perception remain unknown, as scientific research has yet to produce clear findings (Frank, 2023; Gopnik, 2023). The use of DT is anticipated to increase in future generations, raising concerns about possible developmental risks. In this regard, Heffler et al. (2024) detected atypical sensory processes in early digital media experiences, necessitating further research to understand the relationship between screen time and specific sensory-related developmental and behavioral outcomes. The rapid pace of change has led scholars to call for a moratorium to facilitate research, reflection and regulation (Clarke, 2023). Indeed, the identification of activities that both promote and adversely affect the emotional and behavioral wellbeing of adolescents using social networks is crucial (Nicolì et al., 2022).

### 4.4 Limitations

This work has sought to provide a better understanding of the relationship between DT, body image and cultural implications in adolescence, but the framework described is not complete. It was appropriate to point out the benefits that DT can have on the wellbeing of adolescents, about opportunities for social connection or use for creative applications of DT. Another aspect not focused, is the need to equip the young with digital skills to use all the advantages of future digital transformations.

The strength of the study lies in the consideration of body image in a broad development perspective, and role played by the DT on the culture of appearance that strengthens in the adolescent the ideal of beauty characteristic of this growth phase.

## 5 Conclusion

Digital technology not only symbolize the future but have already become pervasive in the lives and interactions of both youth and adults. The impact of these contemporary digital dynamics is particularly significant during adolescence. DT has emphasized a way of communicating among young people “aesthetic” where the power of images and the rigor of representing themselves enhances the story. The use of digital images amplifies the intensity of one’s story, making it more real and even more alive. Adherence to ideal models (thinness ideal) reflects cultural beliefs that increase the sense of belonging. For its prolonged development phase, the adolescent tends to a culture of appearance that exacerbates behaviors related to attention and control of the body, which in turn adhere to beauty standards. The reality that DT and SM propose through idealized images, peer comparison and the stimulus to thinness enhances both cultural beliefs and the critical aspects of this developmental stage. The vulnerability that young people manifest, by engaging in SM, can turn into eating disorders, body aesthetic manipulations, while body dissatisfaction can generate suffering, depressive symptoms as well as self-harm. Particularly young fragile or with body psychological problems are exposed to digital degeneration that uses misleading messages or sites that induce pathological thinness. We also wonder if, in this mechanism, the adolescents are really free to express themselves, to tell their emotions as well as to recognize and approve themselves in SM. What we have described seems to be instead a closed system, poor in experience, in which one is homologated and equal to the others or is crushed if different. Maybe the brains of teenagers are changing too? Let us go to a (near) future where the technology (generative) will be able to create humanized realities. Would we be able to maintain our individuality? We currently have no answers but we can certainly say that the images and representation of themselves that teens do in SM need to be filled with emotional content and real life experiences. This allows a healthy development and a state of wellbeing.

The unprecedented and inadequately understood nature of adolescents’ engagement with DT necessitates further exploration. This involves delving into trends, attitudes, and the repercussions of misinformation on SM and other platforms (Hayawi et al., 2022). Conscious management of social policies and service networks is needed (Kruzan et al., 2022), in order to encourage responsible use of DT among adolescents and to support healthy adaptation and wellbeing. From an academic perspective, it is imperative to enhance our comprehension of the ecological ramifications of DT on human development and to explore methodologies for leveraging technological advancements. This stands as the frontier of knowledge, necessitating continued research efforts.

## References

[ref1] Alipour B. AbbasalizadFarhangi M. Dehghan P. Alipour M. (2015). Body image perception and its association with body mass index and nutrient intakes among female college students aged 18-35 years from Tabriz, Iran. Eat. Weight Disord 20, 465–471. doi: 10.1007/s40519-015-0184-1, PMID: 25701442

[ref2] Amaral A. C. S. Ferreira M. E. C. (2017). Body dissatisfaction and associated factors among Brazilian adolescents: a longitudinal study. Body Image 22, 32–38. doi: 10.1016/j.bodyim.2017.04.006, PMID: 28570920

[ref3] Aniulis E. Sharp G. Thomas N. A. (2021). The ever-changing ideal: the body you want depends on who else you’re looking at. Body Image 36, 218–229. doi: 10.1016/j.bodyim.2020.12.003 33401202

[ref4] Aubrey J. S. Yan K. Gahler H. (2023). Digital technologies and adolescents’ body image. Encycl. Child Adoles. Health 3, 248–259. doi: 10.1016/B978-0-12-818872-9.00156-4

[ref5] Awad M. N. Connors E. H. (2023). Active bystandership by youth in the digital era: microintervention strategies for responding to social media-based microaggressions and cyberbullying. Psychol. Serv. 20, 423–434. doi: 10.1037/ser0000749, PMID: 36951730 PMC10517072

[ref6] Benjamin W. (2022). Das KunstwerkimZeitalter seiner technischenReproduzierbarkeit: DreiStudienzurKunstsoziologie (edition suhrkamp). 36a Edn. Berlin: Suhrkamp Verlag.

[ref7] Blanchard L. Conway-Moore K. Aguiar A. Önal F. Rutter H. Helleve A.. (2023). Associations between social media, adolescent mental health, and diet: a systematic review. Obes. Rev. Off. J. Int. Assoc. Study Obes. 24:e13631. doi: 10.1111/obr.13631 37753597

[ref8] Bordeleau M. Alméras N. Panahi S. Drapeau V. (2023). Body image and lifestyle behaviors in high school adolescents. Child. Basel Switz. 10:1263. doi: 10.3390/children10071263, PMID: 37508760 PMC10377786

[ref9] Borzekowski D. L. G. Bayer A. M. (2005). Body image and media use among adolescents. Adolesc. Med. Clin. 16, 289–313. doi: 10.1016/j.admecli.2005.02.010 16111619

[ref10] Bos M. G. N. Wierenga L. M. Blankenstein N. E. Schreuders E. Tamnes C. K. Crone E. A. (2018). Longitudinal structural brain development and externalizing behavior in adolescence. J. Child Psychol. Psychiatr. 59, 1061–1072. doi: 10.1111/jcpp.12972, PMID: 30255501 PMC6175471

[ref11] Burnette C. B. Kwitowski M. A. Mazzeo S. E. (2017). “I don’t need people to tell me I’m pretty on social media:” a qualitative study of social media and body image in early adolescent girls. Body Image 23, 114–125. doi: 10.1016/j.bodyim.2017.09.001, PMID: 28965052

[ref12] Caceres J. Holley A. (2023). Perils and pitfalls of social media use: cyber bullying in teens/young adults. Prim. Care 50, 37–45. doi: 10.1016/j.pop.2022.10.008 36822726

[ref13] Choukas-Bradley S. Roberts S. R. Maheux A. J. Nesi J. (2022). The perfect storm: a developmental–sociocultural framework for the role of social media in adolescent girls’ body image concerns and mental health. Clin. Child. Fam. Psychol. Rev. 25, 681–701. doi: 10.1007/s10567-022-00404-5 35841501 PMC9287711

[ref14] Clarke L. (2023). Call for AI pause highlights potential dangers. Science 380, 120–121. doi: 10.1126/science.adi2240, PMID: 37053321

[ref15] Czepielewski L. S. (2024). Childhood BMI, adolescent depression, and body dissatisfaction. Lancet Psychiatry 11, 3–4. doi: 10.1016/S2215-0366(23)00407-8, PMID: 38101871

[ref16] Dane A. Bhatia K. (2023). The social media diet: a scoping review to investigate the association between social media, body image and eating disorders amongst young people. PLOS Glob. Public Health 3:e0001091. doi: 10.1371/journal.pgph.0001091, PMID: 36962983 PMC10032524

[ref17] Carvalho G. X. De Nunes A. P. N. Moraes C. L. Da Veiga G. V. (2020). Body image dissatisfaction and associated factors in adolescents. Cienc. SaudeColetiva 25, 2769–2782. doi: 10.1590/1413-81232020257.27452018 32667558

[ref18] De Luca L. Pastore M. Palladino B. E. Reime B. Warth P. Menesini E. (2023). The development of non-suicidal self-injury (NSSI) during adolescence: a systematic review and Bayesian meta-analysis. J. Affect. Disord. 339, 648–659. doi: 10.1016/j.jad.2023.07.091, PMID: 37479039

[ref19] Dienlin T. Johannes N. (2020). The impact of digital technology use on adolescent wellbeing. Dialogues Clin. Neurosci. 22, 135–142. doi: 10.31887/DCNS.2020.22.2/tdienlin, PMID: 32699513 PMC7366938

[ref20] Douglas K. D. Smith K. K. Stewart M. W. Walker J. Mena L. Zhang L. (2023). Exploring parents’ intentions to monitor and mediate adolescent social media use and implications for school nurses. J. Sch. Nurs. Off. Publ. Natl. Assoc. Sch. Nurses 39, 248–261. doi: 10.1177/1059840520983286, PMID: 33375901

[ref21] Festinger L. (1954). A theory of social comparison processes. Hum. Relat. 7, 117–140. doi: 10.1177/001872675400700202

[ref22] Frank M. C. (2023). Baby steps in evaluating the capacities of large language models. Nat Rev Psychol 2, 451–452. doi: 10.1038/s44159-023-00211-x

[ref23] Fredrickson B. L. Roberts T. (1997). Objectification theory: toward understanding women’s lived experiences and mental health risks. Psychol. Women Q. 21, 173–206. doi: 10.1111/j.1471-6402.1997.tb00108.x

[ref24] Gillies D. Christou M. A. Dixon A. C. Featherston O. J. Rapti I. Garcia-Anguita A.. (2018). Prevalence and characteristics of self-harm in adolescents: Meta-analyses of community-based studies 1990-2015. J. Am. Acad. Child Adolesc. Psychiatry 57, 733–741. doi: 10.1016/j.jaac.2018.06.018, PMID: 30274648

[ref25] Gjylbegaj V. (2018). Media effects and body image perceptions on youth in UAE. IJASOS. 4, 415–423. doi: 10.18769/ijasos.455668

[ref26] Goodyear V. (2020). Narrative matters: young people, social media and body image. Child Adolesc. Ment. Health 25, 48–50. doi: 10.1111/camh.12345, PMID: 32285640 PMC7317704

[ref27] Gopnik A. (2023). A very human answer to one of AI’s deepest dilemmas. Washington, DC: Association for Psychological Science.

[ref28] Granic I. Morita H. Scholten H. (2020). Beyond screen time: identity development in the digital age. Psychol. Inq. 31, 195–223. doi: 10.1080/1047840X.2020.1820214

[ref29] Hayawi K. Shahriar S. Serhani M. A. Taleb I. Mathew S. S. (2022). ANTi-vax: a novel twitter dataset for COVID-19 vaccine misinformation detection. Public Health 203, 23–30. doi: 10.1016/j.puhe.2021.11.022, PMID: 35016072 PMC8648668

[ref30] Heffler K. F. Acharya B. Subedi K. Bennett D. S. (2024). Early-life digital media experiences and development of atypical sensory processing. JAMA Pediatr. 178, 266–273. doi: 10.1001/jamapediatrics.2023.5923, PMID: 38190175 PMC10775079

[ref31] Holly L. Wong B. L. H. van Kessel R. Awah I. Agrawal A. Ndili N. (2023). Optimising adolescent wellbeing in a digital age. BMJ 380:e068279. doi: 10.1136/bmj-2021-068279, PMID: 36940933 PMC10019455

[ref32] Holmes A. J. Hollinshead M. O. Roffman J. L. Smoller J. W. Buckner R. L. (2016). Individual differences in cognitive control circuit anatomy link sensation seeking, impulsivity, and substance use. J. Neurosci. 36, 4038–4049. doi: 10.1523/JNEUROSCI.3206-15.2016, PMID: 27053210 PMC4821913

[ref33] Hosseini S. A. Padhy R. K. (2023). Body image distortion. St. Petersburg, FL: StatPearls. 31536191

[ref34] Hummel A. C. Smith A. R. (2015). Ask and you shall receive: desire and receipt of feedback via Facebook predicts disordered eating concerns. Int. J. Eat. Disord. 48, 436–442. doi: 10.1002/eat.22336, PMID: 25060558

[ref35] Jarman H. K. Marques M. D. McLean S. A. Slater A. Paxton S. J. (2021). Social media, body satisfaction and wellbeing among adolescents: a mediation model of appearance-ideal internalization and comparison. Body Image 36, 139–148. doi: 10.1016/j.bodyim.2020.11.005 33285385

[ref36] Jensen M. George M. J. Russell M. R. Odgers C. L. (2019). Young adolescents’ digital technology use and mental health symptoms: little evidence of longitudinal or daily linkages. Clin. Psychol. Sci. 7, 1416–1433. doi: 10.1177/2167702619859336, PMID: 31929951 PMC6953732

[ref37] Jiménez Flores P. Jiménez Cruz A. Bacardi Gascón M. (2017). Insatisfacción con la imagen corporal enniños y adolescentes: revisiónsistemática. Nutr. Hosp. 34, 479–489. doi: 10.20960/nh.455, PMID: 28421808

[ref38] Jones D. C. Vigfusdottir T. H. Lee Y. (2004). Body image and the appearance culture among adolescent girls and boys: an examination of friend conversations, peer criticism, appearance magazines, and the internalization of appearance ideals. J. Adolesc. Res. 19, 323–339. doi: 10.1177/0743558403258847

[ref39] Keeton W. Cash T. Brown T. (1990). Body image or body images?: comparative, multidimensional assessment among college students. J. Pers. Assess. 54, 213–230. doi: 10.1207/s15327752jpa5401&2_21 2313543

[ref40] Khalaf A. M. Alubied A. A. Khalaf A. M. Rifaey A. A. (2023). The impact of social media on the mental health of adolescents and young adults: a systematic review. Cureus 15:e42990. doi: 10.7759/cureus.42990, PMID: 37671234 PMC10476631

[ref41] Kinghorn A. Shanaube K. Toska E. Cluver L. Bekker L.-G. (2018). Defining adolescence: priorities from a global health perspective. Lancet Child Adolesc. Health 2:e10. doi: 10.1016/S2352-4642(18)30096-8 30169271

[ref42] Klonsky E. D. Muehlenkamp J. J. (2007). Self-injury: a research review for the practitioner. J. Clin. Psychol. 63, 1045–1056. doi: 10.1002/jclp.20412, PMID: 17932985

[ref43] Kotiuga J. Vaillancourt-Morel M.-P. Yampolsky M. A. Martin G. M. (2023). Adolescents’ self perceptions: connecting psychosocial competencies to the sexual self-concept. J. Sex Res. 18, 1–11. doi: 10.1080/00224499.2023.2222285 37307397

[ref44] Krogh S. C. (2022). The beautiful and the fit reap the spoils: body image as a condition for the positive effects of electronic media communication on wellbeing among early adolescents. Young 30, 97–115. doi: 10.1177/11033088211009128

[ref45] Kruzan K. P. Williams K. D. A. Meyerhoff J. Yoo D. W. O’Dwyer L. C. De Choudhury M.. (2022). Social media-based interventions for adolescent and young adult mental health: a scoping review. Internet Interv. 30:100578. doi: 10.1016/j.invent.2022.100578 36204674 PMC9530477

[ref46] Lachance J. (2011). L’ adolescence hypermoderne: le nouveau rapport au temps des jeunes. Québec: Presses de l’Université Laval.

[ref47] Lacroix E. Smith A. J. Husain I. A. Orth U. Von Ranson K. M. (2023). Normative body image development: a longitudinal meta-analysis of mean-level change. Body Image 45, 238–264. doi: 10.1016/j.bodyim.2023.03.003, PMID: 36965235

[ref48] Lau-Zhu A. Anderson C. Lister M. (2023). Assessment of digital risks in child and adolescent mental health services: a mixed-method, theory-driven study of clinicians’ experiences and perspectives. Clin. Child Psychol. Psychiatry 28, 255–269. doi: 10.1177/13591045221098896, PMID: 35522928 PMC9893305

[ref49] Le Breton D. (2016). Corps et Adolescence. Bruxelles: Yapaka.be.

[ref50] Magis-Weinberg L. Ballonoff Suleiman A. Dahl R. E. (2021). Context, development, and digital media: implications for very young adolescents in LMICs. Front. Psychol. 12:632713. doi: 10.3389/fpsyg.2021.632713, PMID: 33967899 PMC8097039

[ref51] Mahon C. Seekis V. (2022). Systematic review of digital interventions for adolescent and young adult Women’s body image. Front. Glob. Womens Health 3:832805. doi: 10.3389/fgwh.2022.832805, PMID: 35392118 PMC8982933

[ref52] Marengo D. Longobardi C. Fabris M. A. Settanni M. (2018). Highly-visual social media and internalizing symptoms in adolescence: the mediating role of body image concerns. Comput. Hum. Behav. 82, 63–69. doi: 10.1016/j.chb.2018.01.003

[ref53] Martin M. J. Blozis S. A. Boeninger D. K. Masarik A. S. Conger R. D. (2014). The timing of entry into adult roles and changes in trajectories of problem behaviors during the transition to adulthood. Dev. Psychol. 50, 2473–2484. doi: 10.1037/a0037950 25243329

[ref54] McDonagh J. E. Ambresin A.-E. Boisen K. A. Fonseca H. Jakobsson Kruse P. Meynard A.. (2018). The age of adolescence…and young adulthood. Lancet Child Adolesc. Health 2:e6. doi: 10.1016/S2352-4642(18)30079-8, PMID: 30169304

[ref55] Mesce M. Cerniglia L. Cimino S. (2022). Body image concerns: the impact of digital technologies and psychopathological risks in a normative sample of adolescents. Behav. Sci. 12:255. doi: 10.3390/bs12080255, PMID: 36004826 PMC9405414

[ref56] Moreno M. A. Uhls Y. T. (2019). Applying an affordances approach and a developmental lens to approach adolescent social media use. Digital Health. 5:2055207619826678. doi: 10.1177/2055207619826678, PMID: 30746154 PMC6360465

[ref57] Mushtaq T. Ashraf S. Hameed H. Irfan A. Shahid M. Kanwal R.. (2023). Prevalence of eating disorders and their association with social media addiction among youths. Nutrients 15:4687. doi: 10.3390/nu15214687, PMID: 37960340 PMC10647586

[ref58] Nesi J. Choukas-Bradley S. Prinstein M. J. (2018a). Transformation of adolescent peer relations in the social media context: part 1—a theoretical framework and application to dyadic peer relationships. Clin. Child. Fam. Psychol. Rev. 21, 267–294. doi: 10.1007/s10567-018-0261-x, PMID: 29627907 PMC6435354

[ref59] Nesi J. Choukas-Bradley S. Prinstein M. J. (2018b). Transformation of adolescent peer relations in the social media context: part 2—application to peer group processes and future directions for research. Clin. Child. Fam. Psychol. Rev. 21, 295–319. doi: 10.1007/s10567-018-0262-9, PMID: 29627906 PMC6402323

[ref60] Nicolì I. Spinelli M. Lionetti F. Logrieco M. G. Fasolo M. (2022). Protective and risk activities for emotional and behavioural wellbeing of children and adolescents during the COVID-19 lockdown. Child Care Health Dev. 48, 895–900. doi: 10.1111/cch.13003, PMID: 35297081 PMC9111474

[ref61] Niu S. Yin X. Pan B. Chen H. Dai C. Tong C.. (2024). Understanding comorbidity between non-suicidal self-injury and depressive symptoms in a clinical sample of adolescents: a network analysis. Neuropsychiatr. Dis. Treat. 20, 1–17. doi: 10.2147/NDT.S443454, PMID: 38196800 PMC10773250

[ref62] Norris M. L. Boydell K. M. Pinhas L. Katzman D. K. (2006). Ana and the internet: a review of pro-anorexia websites. Int. J. Eat. Disord. 39, 443–447. doi: 10.1002/eat.20305, PMID: 16721839

[ref63] Odgers C. L. Jensen M. R. (2020). Annual research review: adolescent mental health in the digital age: facts, fears, and future directions. J. Child Psychol. Psychiatry 61, 336–348. doi: 10.1111/jcpp.13190, PMID: 31951670 PMC8221420

[ref64] Ohashi Y. B. Wang S. B. Shingleton R. M. Nock M. K. (2023). Body dissatisfaction, ideals, and identity in the development of disordered eating among adolescent ballet dancers. Int. J. Eat. Disord. 56, 1743–1751. doi: 10.1002/eat.24005, PMID: 37260249 PMC10524937

[ref65] Page M. J. McKenzie J. E. Bossuyt P. M. Boutron I. Hoffmann T. C. Mulrow C. D.. (2021). The PRISMA 2020 statement: an updated guideline for reporting systematic reviews. BMJ 372:n71. doi: 10.1136/bmj.n71, PMID: 33782057 PMC8005924

[ref66] Papageorgiou A. Fisher C. Cross D. (2022). “Why don’t I look like her?” how adolescent girls view social media and its connection to body image. BMC Womens Health 22:261. doi: 10.1186/s12905-022-01845-4, PMID: 35761231 PMC9238066

[ref67] Perloff R. M. (2014). Social media effects on young Women’s body image concerns: theoretical perspectives and an agenda for research. Sex Roles 71, 363–377. doi: 10.1007/s11199-014-0384-6

[ref68] Piran N. (2017). Journeys of embodiment at the intersection of body and culture: The developmental theory of embodiment. London: Academic Press.

[ref69] Revranche M. Biscond M. Husky M. M. (2022). Lien entre usage des réseauxsociauxet image corporelle chez les adolescents: une revue systématique de la littérature. L'Encéphale 48, 206–218. doi: 10.1016/j.encep.2021.08.006, PMID: 34801229

[ref70] Riedel P. Heil M. Bender S. Dippel G. Korb F. M. Smolka M. N.. (2019). Modulating functional connectivity between medial frontopolar cortex and amygdala by inhibitory and excitatory transcranial magnetic stimulation. Hum. Brain Mapp. 40, 4301–4315. doi: 10.1002/hbm.24703, PMID: 31268615 PMC6865431

[ref71] Romeo R. D. (2017). The impact of stress on the structure of the adolescent brain: implications for adolescent mental health. Brain Res. 1654, 185–191. doi: 10.1016/j.brainres.2016.03.021, PMID: 27021951

[ref72] Rosenfeld R. G. Nicodemus B. C. (2003). The transition from adolescence to adult life: physiology of the ‘transition’ phase and its evolutionary basis. Horm. Res. Paediatr. 60, 74–77. doi: 10.1159/000071230, PMID: 12955022

[ref73] Sagrera C. E. Magner J. Temple J. Lawrence R. Magner T. J. Avila-Quintero V. J.. (2022). Social media use and body image issues among adolescents in a vulnerable Louisiana community. Front. Psych. 13:1001336. doi: 10.3389/fpsyt.2022.1001336, PMID: 36405904 PMC9669337

[ref74] Sarwer D. B. Polonsky H. M. (2016). Body image and body contouring procedures. Aesthet. Surg. J. 36, 1039–1047. doi: 10.1093/asj/sjw127 27634782

[ref75] Sawyer S. M. Afifi R. A. Bearinger L. H. Blakemore S.-J. Dick B. Ezeh A. C.. (2012). Adolescence: a foundation for future health. Lancet 379, 1630–1640. doi: 10.1016/S0140-6736(12)60072-5 22538178

[ref76] Sawyer S. M. Azzopardi P. S. Wickremarathne D. Patton G. C. (2018). The age of adolescence. Lancet Child Adolesc. Health 2, 223–228. doi: 10.1016/S2352-4642(18)30022-1 30169257

[ref77] Schuengel C. Van Heerden A. (2023). Editorial: generative artificial intelligence and the ecology of human development. J. Child Psychol. Psychiatry 64, 1261–1263. doi: 10.1111/jcpp.13860, PMID: 37528517 PMC10509500

[ref78] Shen J. Chen J. Tang X. Bao S. (2022). The effects of media and peers on negative body image among Chinese college students: a chained indirect influence model of appearance comparison and internalization of the thin ideal. J. Eat. Disord. 10:49. doi: 10.1186/s40337-022-00575-0, PMID: 35413877 PMC9006462

[ref79] Simons E. I. Noteboom F. Van Furth E. F. (2024). Pro-anorexia coaches prey on individuals with eating disorders. Int. J. Eat. Disord. 57, 124–131. doi: 10.1002/eat.24074 37906085

[ref80] Soares Filho L. C. Batista R. F. L. Cardoso V. C. Simões V. M. F. Santos A. M. Coelho S. J. D. D. A. C.. (2021). Body image dissatisfaction and symptoms of depression disorder in adolescents. Braz. J. Med. Biol. Res. 54:e10397. doi: 10.1590/1414-431x202010397 PMC7727113 33295537

[ref81] Sonmez M. Esiyok E. (2023). The effect of social media usage, appearance-related social media pressure and body mass index on body appreciation of cosmetic procedure patients. Aesth. Plast. Surg. 47, 2711–2718. doi: 10.1007/s00266-023-03654-y, PMID: 37737876

[ref82] Subrahmanyam K. Šmahel D. (2011). Digital youth: the role of media in development. Cham: Springer.

[ref83] Thompson J. K. Heinberg L. J. Altabe M. Tantleff-Dunn S. (1999). Exacting beauty: Theory, assessment, and treatment of body image disturbance. Washington, DC: American Psychological Association.

[ref84] Tylka T. L. Wood-Barcalow N. L. (2015). What is and what is not positive body image? Conceptual foundations and construct definition. Body Image 14, 118–129. doi: 10.1016/j.bodyim.2015.04.001, PMID: 25921657

[ref85] Valkenburg P. M. Peter J. (2011). Online communication among adolescents: an integrated model of its attraction, opportunities, and risks. J. Adolesc. Health 48, 121–127. doi: 10.1016/j.jadohealth.2010.08.020, PMID: 21257109

[ref86] Vall-Roqué H. Andrés A. Saldaña C. (2021). The impact of COVID-19 lockdown on social network sites use, body image disturbances and self-esteem among adolescent and young women. Prog. Neuro-Psychopharmacol. Biol. Psychiatry 110:110293. doi: 10.1016/j.pnpbp.2021.110293, PMID: 33662532 PMC8569938

[ref87] Vankerckhoven L. Raemen L. Claes L. Eggermont S. Palmeroni N. Luyckx K. (2023). Identity formation, body image, and body-related symptoms: developmental trajectories and associations throughout adolescence. J. Youth Adolesc. 52, 651–669. doi: 10.1007/s10964-022-01717-y 36484894 PMC9735114

[ref88] Vogels E. Gelles-Watnick R. Massarat N. (2022). Teens, social media and technology. Washington, DC: Pew Research Center.

[ref89] Weigle P. E. Shafi R. M. A. (2024). Social media and youth mental health. Curr. Psychiatry Rep. 26, 1–8. doi: 10.1007/s11920-023-01478-w 38103128

